# Concurrent EGFR Mutation and ALK Translocation in Non-Small Cell Lung Cancer

**DOI:** 10.7759/cureus.513

**Published:** 2016-02-26

**Authors:** Randy F Sweis, Sachdev Thomas, Bruce Bank, Paul Fishkin, Colin Mooney, Ravi Salgia

**Affiliations:** 1 Hematology/Oncology and Clinical Pharmacology, University of Chicago; 2 VA Central California Health Care System/UCSF-Fresno, University of California San Francisco; 3 Medical Oncology, Northwest Oncology and Hematology; 4 Medical Oncology, Illinois Cancer Care; 5 Internal Medicine, Medical College of Wisconsin; 6 Medical Oncology and Therapeutics Research, City of Hope

**Keywords:** personalized medicine, genomics, molecular subtypes, non-small-cell lung cancer, alk-positive adenocarcinoma, egfr mutations in lung adenocarcinoma

## Abstract

Epidermal growth factor receptor (EGFR) mutations and anaplastic large-cell lymphoma kinase (ALK) rearrangements are now routine biomarkers that have been incorporated into the practice of managing non-small cell lung cancer (NSCLC). Historically, the two molecular alterations have been viewed as mutually exclusive, but recent identified cases suggest otherwise. In this report, we describe cases of lung cancer with concurrent EGFR mutation and ALK rearrangement and identify their clinical characteristics.

Non-small cell lung cancer patients with multiple molecular alterations were retrospectively analyzed from an academic referral center from 2011–2013. An additional review was conducted of reported cases with dual alterations. Four cases of NSCLC with alterations in both EGFR and ALK were identified and evaluated with 16 published cases for a total of 20 cases. The age of patients ranged from 37 to 77 years. Nine patients were never smokers. The disease control rates in patients treated with EGFR inhibitors and ALK inhibitors were 46% (6/13) and 71% (5/7), respectively.

This series highlights the importance of comprehensive molecular profiling of newly diagnosed lung cancer, as NSCLC may be driven by concurrent molecular alterations. EGFR- and ALK-targeted therapies appear to have modest activity in patients with tumors possessing both alterations. Dual-altered NSCLC patients may have distinct clinical characteristics warranting further study. Combination targeted therapy or novel multi-targeted tyrosine kinase inhibitors may prove important in these patients, though necessary studies remain ongoing.

## Introduction

The detection and targeting of genetic alterations in lung cancer has transformed our approach to therapy over the past decade. Somatic gene mutations or rearrangements in specific “driver genes” are thought to result in oncogenic transformation and tumor growth. This paradigm shift has been exemplified by the discovery of activating mutations in the epidermal growth factor receptor (EGFR) and rearrangements of the anaplastic large-cell lymphoma kinase (ALK) gene. Clinical guidelines have now incorporated molecular testing and the use of drugs targeting those genes [1]. Inhibitors of EGFR, such as erlotinib and gefitinib have demonstrated clinical activity in tumors whose growth depends on constitutive activation of EGFR [2-4]. Similarly, the echinoderm microtubule-associated protein-like 4-anaplastic lymphoma kinase (EML4-ALK) fusion gene resulting from the chromosome inversion inv(2)(p21;p23) was identified as another independent driver of lung cancer oncogenesis [5]. Targeting of this abnormality with crizotonib has been clinically successful [6]. Up to 20% of lung cancers carry one of these two alterations, with higher percentages in light- or never-smokers.

Despite the clinical activity observed with drugs targeting EGFR or ALK, tumors eventually acquire resistance and overall survival remains poor. There remains a continued need to improve our understanding of the complex biology that drives lung tumors and advance existing therapeutic tactics. Historically, EGFR and ALK alterations have been viewed as independent, mutually exclusive events [7-8]. Proposed algorithms for testing are variable [1]. One approach tests for EGFR-activating mutations followed by ALK testing only if EGFR is wild-type. Another approach is to test for KRAS mutations first then halt further testing if mutated. However, recent reports of patients with alterations in both EGFR and ALK have surfaced [9-18]. Various responses to existing targeted drugs have been reported. In this report we present a series of patients presenting with dual alterations in EGFR and ALK and propose an updated methodology of testing and treatment. Informed patient consent was obtained for this study. 

## Case presentation

The characteristics of patients in this series and from prior reports are summarized jointly in Table 1. All patients were found to have alterations in EGFR and ALK.

**Table 1:** Characteristics of patients with concurrent EGFR and ALK alterations *=not reported, ^1^Secondary mutation on crizitonib, NT=not treated, GNC=gene copy number

Citation	Age	Sex	Smoking	Stage	EGFR Alteration	EGFR-TKI		ALK Variant	ALK-TKI	
						Response	Months		Response	Months
16	72	F	Never	IV	Exon19 deletion	PR	7	1	NT	
12	65	F	Never	IV	Exon19 deletion	CR	25	*	NT	
13	48	M	Never	IV	Exon19 deletion	PD	2	*	NT	
18	*	F	Never	*	Exon19 deletion	*		3b	NT	
15	39	M	Light	IV	L858R	PD	1	3b	NT	
10	55	F	Never	IV	Exon19 deletion	SD	3	2	SD	4
9	77	F	Never	*	L861Q	PD	2	↑GCN	SD	4
11	*	*	*	*	Exon19 deletion	*		*	*	
14	*	*	*	*	L858R	PR	9	*	*	
14	*	*	*	*	Exon19 deletion	PR	5+	*	*	
14	*	*	*	*	A767_V769dupASV	NT		*	*	
17	73	M	15 p/y	IV	Exon19 deletion	PD	<1	*	PR	9+
17	*	*	*	IIB	Exon19 deletion	NT		*	NT	
17	*	*	*	IIIA	L718P	NT		*	NT	
17	*	*	*	IA	L858R	NT		*	NT	
21	61	M	Never	IV	L862R^1^	NT		*	PR	8
Case 1	37	M	Never	IV	Exon23 polymorphism	PR	12	*	SD	9
Case 2	57	F	15 p/y	IV	L861Q	PR	2	*	NT	-
Case 3	66	F	Never	IV	Exon19 deletion	PD	2	*	PD	1
Case 4	52	M	30 p/y	IV	L858R	PD	2	*	PD	<1

Patient One was a South East Asian-American male diagnosed with stage IV adenocarcinoma of the lung with a malignant pleural effusion in November, 2011 at age 37. He was found to have an ALK rearrangement and an EGFR exon 23 polymorphism. He was a never-smoker. His first-line therapy was carboplatin, paclitaxel, and bevacizumab for six cycles. He had a partial response and was next treated with erlotinib and bevacizumab for a total of 12 months. At progression, he was treated with pemetrexed for three months. Eventually his cancer progressed again, and he was treated with crizotinib for nine months with stable disease. Ultimately, he had progression of disease in his brain and died.

Patient Two was a 57-year-old female who was diagnosed with stage IV adenocarcinoma of the lung after presenting with weight loss and shortness of breath in November, 2011. She had a 15 pack-year history of cigarette smoking, but quit 10 months prior to presentation. She was found to have a lung mass and pleural effusion that was positive for adenocarcinoma. An ALK rearrangement and EGFR translocation (L861Q, exon 21) were identified. She was treated with carboplatin and pemetrexed and had a partial response after two cycles. She completed six cycles, but a follow up CT scan showed progression of the disease, so she was treated with second-line docetaxel. She did not respond to the drug and was placed on a clinical trial with a second generation ALK inhibitor. Unfortunately, the disease progressed rapidly and she died before starting that drug.

Patient Three presented in May, 2013 at age 66 with a new diagnosis of stage IV adenocarcinoma of the lung after presenting with persistent spastic coughing and chest pain. She was a never-smoker. Imaging revealed widespread metastases including in the brain and several vertebrae. She was found to have an ALK rearrangement and EGFR exon 19 deletion. She was treated with erlotonib and had progression of disease after two months. Next, she was treated with crizotinib, but the diesease progressed after one month and she died shortly thereafter.

Patient Four was diagnosed with stage IV adenocarcinoma of the lung at age 52 in November, 2012. He had a 30 pack-year smoking history, but quit seven years prior. He presented with rib pain and was found to have a lung mass along with numerous metastases including in the rib and lumbar spine. A biopsy of the lung mass revealed adenocarcinoma with EML4-ALK translocation as well as EGFR mutation (L858R, exon 21). He was initially treated with crizitonib. While his primary lung mass decreased in size, a positron emission tomography (PET) scan showed overall progression of the disease primarily in bone. He was then treated with four cycles of carboplatin/pemetrexed. Though he partially responded, chemotherapy was poorly tolerated. When his disease progressed, he was switched to erlotonib. Repeat imaging two months later showed further progression of the disease. At that point he elected for no further therapy.

## Discussion

**Figure 1:**
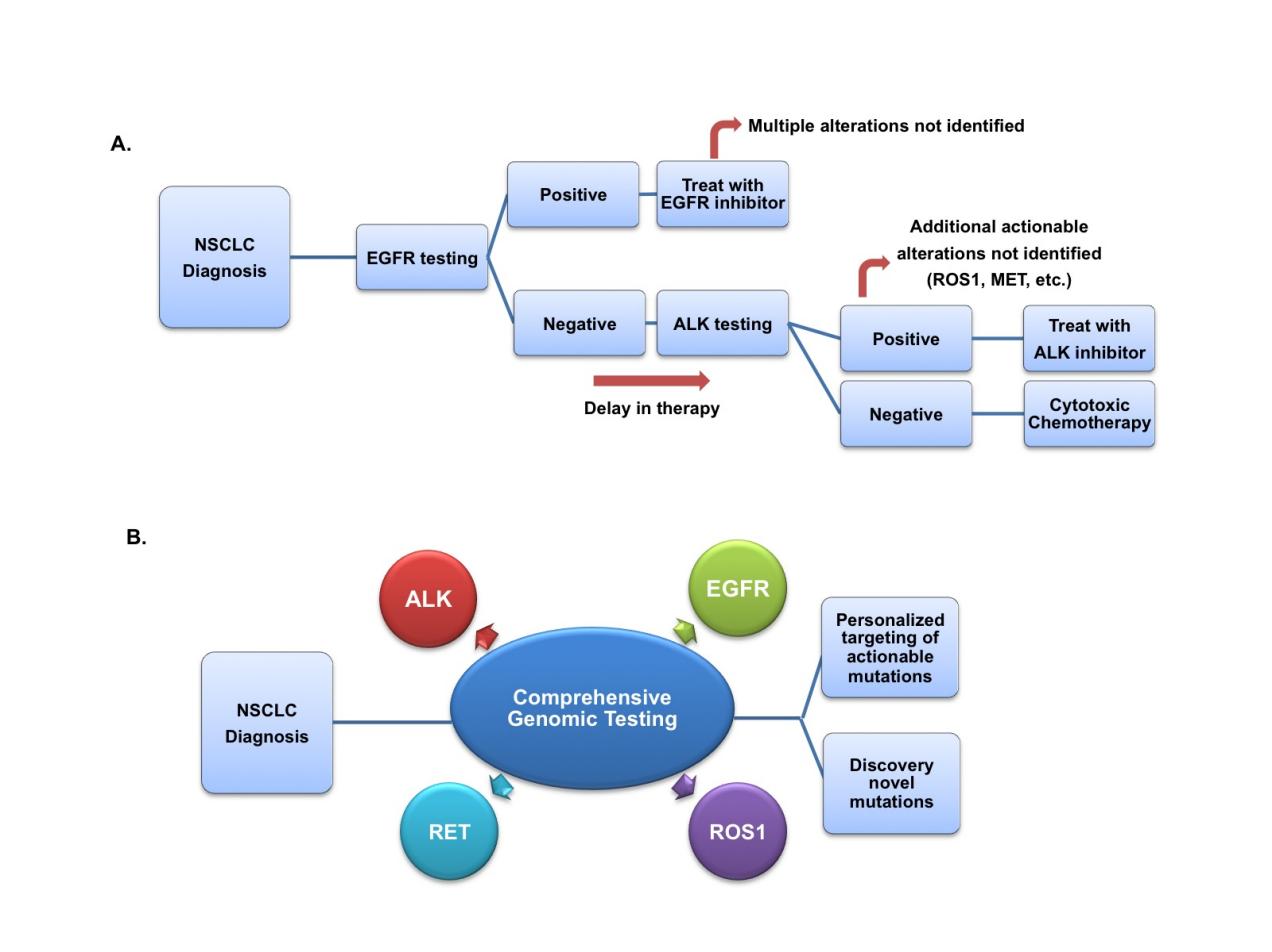
Therapeutic algorithms for molecular diagnosis and treatment of NSCLC (A) Historical molecular diagnostic algorithm, (B) Proposed updated methodology

EGFR and ALK molecular alterations were discovered independently and have been presumed to be mutually exclusive cancer drivers. Recently published guidelines recommend that EGFR testing be prioritized over other molecular markers [1]. This case series describes four patients who presented with concurrent alterations in both EGFR and ALK. Testing for a single molecular alteration or using sequential testing precludes identification of this group of dual-altered tumors, which has distinct clinical characteristics. Furthermore, up to 3% of lung cancer patients have more than one molecular alteration [19]. Comprehensive genomic analysis (i.e. next generation sequencing) of lung tumors on initial diagnosis should be considered to better categorize patients into prognostic groups and develop therapeutic algorithms (Figure 1). The cost of next-generation sequencing continues to decline and is approaching the cost of performing all indicated biomarker tests individually.

The age of presentation varied widely from 37 to 66 and there was no gender predominance, both features consistent with prior reported cases. Nearly all patients were never- or light-smokers. Dual-altered lung adenocarcinoma with multiple somatic mutations may result in a more aggressive phenotype with an earlier propensity to metastasize. All patients in this series presented with stage IV disease, and lytic bony metastases were predominant. Recent data reported from the Lung Cancer Mutation Consortium suggest that the survival rate was worse in patients with more than one molecular alteration than in patients with zero or one alteration [19].

The optimal treatment approach and sequence for dual-altered lung cancer requires further investigation. The majority of reported dual-altered lung cancer patients were treated with a platinum doublet as first-line therapy. All had a short interval of response or none at all. In many cases, this approach had been chosen based on the lack of approved targeted agents at the time of presentation. Though pemetrexed has reported efficacy in cases with ALK-EML4 translocation [20], this was not observed in dual-altered patients. Patients with a driver mutation have better survival when treated with targeted therapy [19]. Therefore we recommend targeted agents as first-line therapy in dual-altered patients.

Exon 19 deletions and exon 21 L858R mutations account for 90% of reported EGFR mutations. In this series, the specific mutations in EGFR varied. In fact, one patient had an L861Q mutation, which has not been previously reported in ALK-EML4 positive patients. Another patient had an exon 23 polymorphism, which also has not been published. The correlation between the sensitivity of currently approved EGFR inhibitors with less common mutations is not as well established. In total, 7 out of 13 patients treated with erlotinib had disease control, which is less than expected in patients with isolated EGFR mutations [4]. All of the four previously described patients treated with an ALK inhibitor showed disease control. Although in this series, only 1 out of 3 patients responded to ALK inhibition.

The heterogeneity of the alterations in this series suggests an absence of clonality. This population may not represent a distinct biologic subgroup and is more likely a combination of random de novo somatic mutations. In some reported cases, EGFR mutation was acquired during treatment with crizitonib [21]. In vitro data suggest that the addition of EGFR inhibition reverses this mode of secondary resistance [11,14]. However, clinical evidence supporting concomitant use of currently available EGFR and ALK inhibitors is not yet available and may be limited by toxicity.

In patients with dual mutations, the mechanism of primary resistance to targeted therapies may be unique and is yet to be elucidated. At least one of the more recently discovered molecular drivers such as ROS 1, C-MET/RON, RET, or PIK3CA may play a role. At this time, there is insufficient data to guide selection of EGFR versus ALK inhibition as initial therapy, and both approaches remain justifiable. However, combined data from this series and prior reports indicate a higher response rate with ALK-targeted therapy as compared with EGFR-targeted therapy. Clinical trials using combination targeted therapy are still ongoing as are studies with next-generation irreversible and/or multi-targeted agents. Treatment strategies for dual-altered patients will likely evolve rapidly in the coming years. Novel multi-targeted tyrosine kinase inhibitors may prove beneficial in the treatment of this group of patients.

## Conclusions

Non-small cell lung cancer may be driven by multiple molecular alterations. We report four cases with concurrent EGFR mutation and ALK translocation. Such cancers may have unique characteristics warranting further study. Comprehensive genomic profiling is critical for NSCLC, as novel therapeutic strategies are being devised including combination targeted therapy or multi-targeted tyrosine kinase inhibitors.
